# Primary breast osteosarcoma in a patient previously treated for ipsilateral invasive ductal carcinoma: An unusual case report with clinical and genomic features

**DOI:** 10.3389/fonc.2022.1013653

**Published:** 2023-01-23

**Authors:** Siji Zhu, Haoyu Wang, Lin Lin, Xiaochun Fei, Jiayi Wu

**Affiliations:** ^1^ Department of General Surgery, Comprehensive Breast Health Center, Ruijin Hospital Affiliated to Shanghai Jiao Tong University School of Medicine, Shanghai, China; ^2^ Department of Clinical Laboratory, Ruijin Hospital Affiliated to Shanghai Jiao Tong University School of Medicine, Shanghai, China; ^3^ Department of Pathology, Ruijin Hospital Affiliated to Shanghai Jiao Tong University School of Medicine, Shanghai, China

**Keywords:** breast malignancy, extraskeletal osteosarcoma, primary breast osteosarcoma, genomic profile, molecular therapy

## Abstract

Primary breast osteosarcoma is a rare subtype of breast malignancy with limited clinical evidence, inadequate biological understanding, and unmet treatment consensus. Here, we report an unusual case of primary breast osteosarcoma developing in the same quadrant of the breast 2 years after initial dissection and radiation of invasive ductal carcinoma. Thorough evaluations of imaging and pathology were conducted while genomic alterations of both primary and secondary tumors, as well as peripheral blood samples, were explored through the next-generation sequencing technique. A comprehensive review of the current literature was also performed on this rare malignancy.

## Introduction

Primary breast osteosarcoma (PBOS) is an extremely rare subtype of breast sarcoma, with published data being limited to case reports and small series (1). Given the rarity of this tumor and divergence concerning its histogenesis, diagnosis, treatment, and prognosis, there is no common consensus regarding the management of this specific kind of malignancy. Therefore, reporting each case and its challenges could be helpful to expand the available knowledge base in the hopes of eventually improving patient care. Here, we report the case of a patient who developed a primary osteogenic sarcoma of the breast 2 years after being treated by surgery and radiation for invasive carcinoma of the ipsilateral breast. Genomic sequencing was conducted to further explore the molecular characteristics of this unusual malignancy.

## Case description

### Patient history and presentation

A 42-year-old woman presented with a 3-week history of a painless, mobile, firm, 2.5-cm lump in the lower outer quadrant of the left breast without axillary lymphadenopathy. No evidence of nipple retraction or discharge was observed. The physical exam of the contralateral breast was unremarkable.

She was already known, having been treated 2 years previously for a left invasive ductal carcinoma (lower outer quadrant, triple negative, grade 3, and Ki-67 70%) without nodal involvement (pT1bN0M0, stage IA). At that time, she underwent lumpectomy and sentinel lymph node biopsy followed by anthracycline/taxane-based adjuvant chemotherapy and radiotherapy (40 Gy in 15 fractions prescribed to ipsilateral whole breast with a 10-Gy boost in four fractions to the tumor bed).

**Figure 1:**
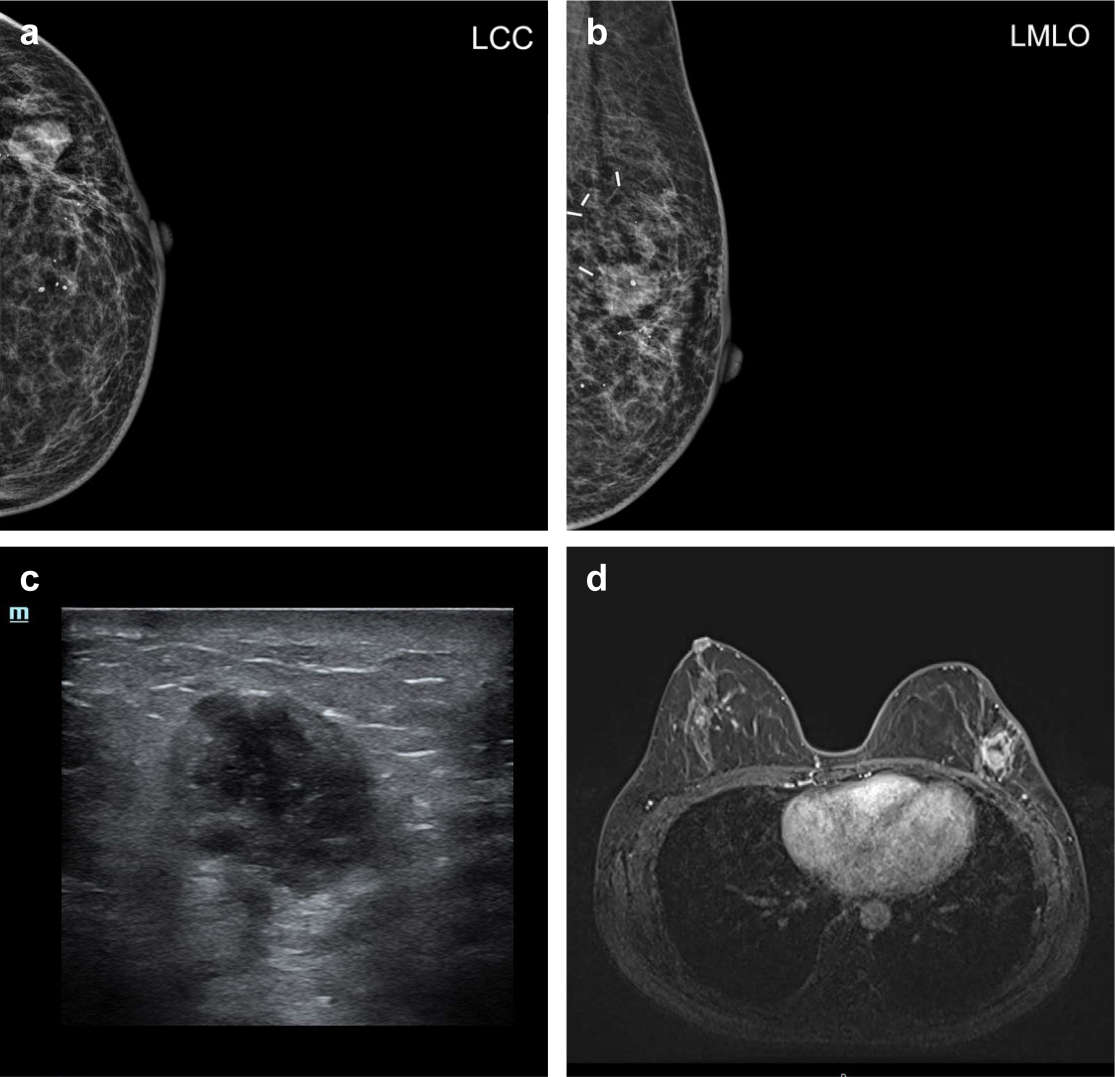
Radiology. **(A)** Left craniocaudal (LCC) view and **(B)** left mediolateral oblique (LMLO) view of mammography. **(C)** Representative ultrasound imaging of a breast lesion. **(D)** Representative MRI imaging of a breast lesion.

At first, the new symptomatic swelling presenting in the same quadrant 2 years after primary treatment was highly suspicious of local recurrence. The mammography and ultrasonography revealed an irregular, bulky mass with a lobulated border in the lateral part of the left breast (**Figures 1A–C**). On MRI, there was a 3-cm mass in the lateral part of the left breast with a high signal intensity at the periphery of the tumor (**Figure 1D**). The diagnosis and treatment timeline are demonstrated in **Supplementary Figure S1**.

### Pathological evaluation and diagnoses

The initial core needle biopsy suggested a spindle-cell malignant tumor with osteoid matrix and necrosis, and therefore, an excisional biopsy was then performed. On gross examination, the specimen was 50 mm × 40 mm × 30 mm and contained a medium-to-firm texture nodular lesion measuring 20 mm in maximum dimension and surrounded by fibro-fatty tissue.

**Figure 2:**
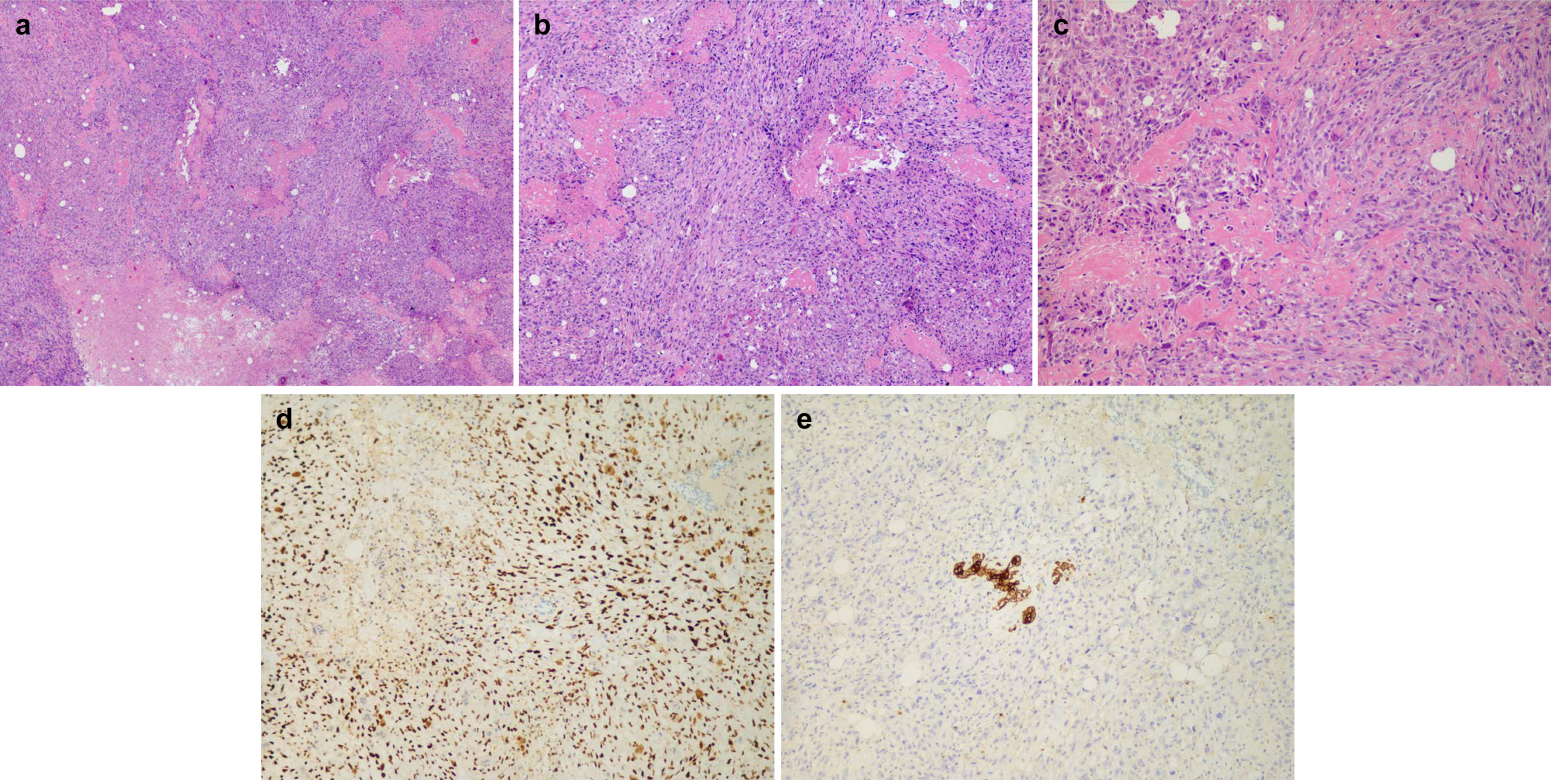
Histology: **(A)** ×25 magnification, **(B)** ×50 magnification, and **(C)** ×100 magnification of H&E staining for representative osteoid matrix and necrosis. **(D)** Immunohistochemistry (IHC) staining of SATB in representative tumor areas. **(E)** IHC staining of AE1/3 in representative tumor areas.

Microscopically, the lesion was composed of abundant pleomorphic, spindle, and oval cells with infiltrative growth patterns. The tumor cells revealed eosinophilic cytoplasm, prominent nucleoli, and a high mitotic index. Osteoid matrix and necrosis were frequently seen at the periphery of the tumor (**Figures 2A–C**). No evidence of infiltrating ductal carcinoma or ductal carcinoma *in situ* was observed. The following immunohistochemistry (IHC) results were obtained: Cytokeratin AE1/AE3 (AE1/AE3) (−), cluster of differentiation 56 (CD56) (focal+), special AT-rich sequence-binding protein (SATB) (+), murine double minute2 (MDM2) (+), smooth muscle actin (SMA) (partial+), Ki-67 (80%), cytokeratin 7 (CK7) (−), estrogen receptor (ER) (−), progesterone receptor (PR) (−), human epidermal growth factor receptor 2 (Her2) (0), cluster of differentiation 34 (CD34) (−), and S-100 (−) (**Figures 2D, E**). The negativity of AE1/AE3, an epithelial marker, reconfirmed the lack of an epithelial component. On the other hand, SATB was proven to be involved in the process of osteoblastic differentiation, which also authenticated our pathological prognosis as PBOS (2).

### Genomic panel

To validate the pathological diagnosis of PBOS and explore the molecular connections between the PBOS and the previous invasive ductal carcinoma (IDC), a commercially targeted NGS was performed on both primary and secondary tumor slices for somatic mutations and peripheral blood samples for germline gene variants. A total of 421 gene variants related to target therapy, immune therapy, chemotherapy response, and genetic predisposition among breast cancer patients were included in the genomic panel. No germline variations were found for this patient. Several copy number variant (CNV) events were identified in the PBOS sample, including CN gains of *FGFR1* and CN loss of *CDKN2A* and *TSC2*. Regarding somatic mutations, *PIK3CA* p.H1047R, *PTEN* p.V275G, *TP53* p.T81Nfs*64, and *TSC2* p.C728Lfs*34 were detected, with the highest variant allele frequency (VAF) of 59.22% happening in the *PIK3CA* mutation. Interestingly, somatic *PIK3CA* p.H1047R, *PTEN* p.V275G, and *TP53* p.T81Nfs*64 were repeatedly detected in both PBOS and IDC tumor samples. On the other hand, somatic *EGFR* p.E709K was uniquely found in the IDC sample.

A written and signed informed consent was obtained from the patients and presented as supplementary material.

### Medical management

A CT of the chest, abdomen, and pelvis did not identify any metastases. An 18F-FDG PET/CT scanning was undertaken, and no evidence of a distant lesion or primary osteosarcoma arising from bone was detected, indicating that the breast lesion was primary osteosarcoma.

As per our institute routine, the patient was discussed in a multidisciplinary team (MDT), and a skin-sparing mastectomy followed by immediate breast reconstruction with a deep inferior epigastric perforator (DIEP) flap was achieved for her. According to the MDT’s opinion, axillary lymph node sampling was not performed. No residual lesion was identified histologically. No adjuvant treatment was recommended. The patient is under regular follow-up right now. The latest follow-up was done on 25 November 2022, and the patient is still alive.

## Discussion

### Epidemiology

Primary breast sarcomas comprise only 0.0006%–1% of all breast malignancies, and PBOS is far less common, accounting for approximately 4%–12.5% of primary breast sarcomas (1, 3). To our knowledge, only approximately 150 cases have been published in the literature (4). Additionally, a study from Nottingham University showed that the vast majority of reported PBOS were actually some variants of metaplastic breast carcinoma due to the lack of a comprehensive histological and IHC evaluation (5).

### Clinical presentation

There is a wide range of onset ages of PBOS in the literature, ranging from range from 16 to 96 years old (6, 7). Meanwhile, in contrast to skeletal osteosarcomas, which tend to present at a younger age, three relatively large series published in the 1990s from MD Anderson, Mayo Clinic, and Armed Forces Institute of Pathology in Washington, DC, reported the same major age span of 40–60 years (1, 3, 8).

PBOS typically presents as a hard, painless, palpable mass with no attendant evidence of nipple discharge or retraction, nor axillary lymphadenopathy (1, 9). Similar to malignant phyllodes tumors, PBOS exhibits rapid growth, which may account for the large average size (4.6 cm) at presentation (10).

### Predisposing factors

A prior history of burns, trauma, or even a foreign body has been reported in some cases of PBOS (1, 6). In addition, some cases presented with a history of epithelial breast cancer on the same side or contralateral side (11, 12). Of note, some patients have been reported to have developed PBOS after undergoing radiotherapy (13, 14). It has been reported in previous literatures that the interval of developing radiotherapy-induced sarcoma (RIS) was more than 10 years (15, 16). In this case, the patient developed PBOS after having surgery and radiotherapy for breast cancer, with a relatively shorter latency period of only 2 years. On the other hand, chemotherapy may also contribute to the newly developed sarcoma. In a retrospective cohort study from the SEER database, it was found that alkylating agents were associated with a higher risk of developing sarcomas with a RR of 7.7 (17). Additionally, another cohort study found that chemotherapy shortened the median interval of RIS development from 14 to 8 years compared with chemotherapy-free patients. Strikingly, alkylating agents and anthracyclines, which generate DNA double-strand breaks, have been reported to significantly shorten the latency of radiotherapy-induced sarcomas (18). The history of ipsilateral breast cancer, the trauma of previous surgery, radiation exposure, and chemotherapy agents may all be the risk factors for developing PBOS. However, there was no conclusive evidence of the driving carcinogenesis factors. Hence, the tumor could be described as “postradiation” rather than “radiation-associated.”

### Imaging and pathological diagnostic workup

The workup of the diagnosis for PBOS included imaging evaluation and pathological diagnosis. For a breast lump, mammography and ultrasound were most commonly used. However, the mammographic and ultrasonic findings of PBOS would present similarly to benign lesions such as fibroadenoma, which may lead to misdiagnosis (19). Furthermore, before labeling them as a PBOS, other neighbors’ origins such as underlying ribs, sternum, and even the pectoralis muscle, as well as metastatic osteosarcoma from the bone, must be ruled out. Hence, in the case of evidence for PBOS on a core needle biopsy, in addition to the routine workup for breast cancer, some other evaluations, such as CT, MRI, skeletal scintigraphy, or PET/CT, may be included. Dynamic contrast-enhanced MRI could be used for additional evaluation and information (20). CT and PET/CT can be useful to identify distant metastases while also playing important roles in ruling out primary skeletal osteosarcomas together with skeletal scintigraphy (9, 21).

Concerning pathological diagnosis, the utility of core needle biopsy in the preoperative workup of patients with PBOS has been described in some literatures (22–24). However, as a case reported in 2019 described, a core needle biopsy from a calcified breast lesion was initially misdiagnosed as benign metaplastic ossification, and only after lumpectomy was the breast osteosarcoma identified, demonstrating the importance of excision sampling (25). Numerous tumors of the breast-producing cartilage, osteoid, and bone, such as metaplastic carcinoma and malignant phyllodes tumors with osteosarcomatous differentiation, should be taken into consideration in differential diagnoses (5, 6, 24). In this case, given the history of ipsilateral breast cancer, it was essential to identify whether it was an ipsilateral carcinoma recurrence. The absence of epithelial cells on extensive immunohistochemistry could rule out the diagnosis of metaplastic carcinoma and, logically, the local recurrence of previous breast cancer. Therefore, given the complexity of PBOS, confirmation of a consistent morphologic pattern required sampling of the whole lesion and extensive sectioning.

### Treatment

Due to the rarity of breast osteosarcomas, there is no general and comprehensive consensus on the management of PBOS. As the literatures reported, PBOS tends to be similar to sarcomas arising at other locations, presenting local aggression with blood spread rather than lymphatic spread (1, 23). Achieving a negative margin either with wide local excision or a simple mastectomy without axillary assessment is likely to be the most judicious option for the majority of patients (4, 10, 26, 27). Of note, the pathological diagnosis before definite surgery was quite important to guide axillary management.

Aside from surgical principles, the benefits of chemotherapy and radiation for PBOS have also been discussed in many literatures (8, 19, 28–30). Based on limited published works, the role of chemotherapy is uncertain with differing regimens and outcomes, and radiotherapy does not appear to improve outcomes. However, due to the unfavorable prognosis reported, chemotherapy and chest wall irradiation have been suggested by some authors to reduce the risk of recurrence, particularly for patients with a tumor size of more than 5 cm (28, 29, 31).

In our opinion, an appropriate approach, including surgery and administration of chemotherapy or radiotherapy, must be balanced against the consequences of these treatments on a case-by-case basis. In this case, taking the relatively young age (42 years old), small tumor size (2 cm), history of breast cancer with chemo/radiotherapy, and patient’s opinion into consideration, the multidisciplinary team finally suggested the radical surgery as mastectomy followed by immediate breast reconstruction, without axillary assessment or adjuvant therapy.

### Genomic information and histogenesis exploration

Despite a comprehensive understanding of the genomic landscapes of both breast cancer and osteosarcoma (32), little is known about the genomic features and histological origins of PBOS due to its extremely rare morbidity. According to previous literatures, extraskeletal osteosarcoma (ESOSA) generally shared similarities in pathological and molecular characteristics with conventional adolescent osteosarcoma (33). In our case, a frameshift mutation of *TP53* (p.T81Nfs*64) indicated a total loss of function; a missense mutation of another tumor suppressor, *PTEN*, was also detected. Both of these mutations were typical genomic alteration events in conventional osteosarcoma, accounting for 80% and 44% of the cases, respectively (34). Moreover, CNV events including *FGFR1* gain and *CDKN2A* loss were commonly identified in osteosarcoma, as previously reported, proving the pathological diagnosis of PBOS from a molecular aspect. Nevertheless, despite considerable alterations in phosphatidylinositol 3-kinase/mammalian target of the rapamycin (PI3K/mTOR) pathway (35), variants including *PIK3CA* mutation, *TSC2* mutation, and *TSC2* loss were extremely rare in conventional osteosarcoma (34). For example, the *PIK3CA* mutation was found in approximately 3% of sarcomas according to TCGA database (36) and was barely been reported until its first discovery in 2012 (35, 37). Interestingly, it has been reported that ESOSA may display unique genomic alterations compared with conventional osteosarcomas, especially with more mutations in *PIK3CA* and PI3K/mTOR pathways (33). Moreover, a patient-derived cell line of PBOS was recently established and validated by NGS genomic testing. A somatic mutation of *PIK3CA* p.H1047R was also detected, indicating that ESOSA, especially PBOS, may harbor actionable genomic alterations in *PIK3CA* and PI3K/mTOR pathways (38).

To investigate the potential histogenesis and evolution of our case, the genomic profiles were also compared between the PBOS and initial IDC samples. Notably, despite distinctive histopathological features, somatic *PIK3CA* p.H1047R, *PTEN* p.V275G, and *TP53* p.T81Nfs*64 were repeatedly detected in both PBOS and IDC tumor samples. The high genomic similarity made us wonder whether these two chronological malignancies had the same origins in tumorigenesis. Shared mutations may indicate a predominant clone, which could be identified as a common ancestor, or cancer stem cells (CSCs), during early tumor formation. Multipotent CSCs could then differentiate into multiple cell lineages and passively accumulate branch mutations under external pressures such as radiation and trauma (39). Several studies have delivered evidence or opinions supportive of our hypothesis (5, 40, 41). It has been reported through an animal experiment that canine mammary osteosarcomas could originate from a pluripotent mammary stem cell (40). Literature reviews and case reports also offered evidence that PBOS may be epithelial in origin and underwent an ossifying evolution process (5, 41). Still, current evidence is not valid enough to elucidate the histogenesis of PBOS.

On the other hand, could the newly diagnosed PBOS be a metaplastic recurrence of primary Triple-negative breast cancer (TNBC)? To explore this question, the genomic documents of PBOS were compared to those of metastatic TNBC in previous literatures. Although breast cancer could develop new genomic alterations during metastatic progression, several studies have found that recurrent TNBC shared similar genomic profiles compared with matched primary TNBC (42, 43). *TP53* mutation was mostly detected (~80%) in both primary and metastatic TNBC, while *PTEN* mutation occurred in 8% of advanced TNBC, which were both detected in our case. Thus, it is really hard to differentiate PBOS from a TNBC recurrence. Nevertheless, when we look at the intrinsic subtype of TNBC defined by Lehmann et al., it is shown that *PIK3CA*, *PTEN*, and PI3K/mTOR pathways are mostly altered in the mesenchymal-like subtype. TNBC with mesenchymal-like features had genomic similarity with metaplastic breast cancers, which harbored lineage plasticity, including cartilaginous differentiation (44). Taken together, the genomic profile of paired tumors may indicate that the PBOS originated from the primary IDC and progressed from metaplastic components; however, more solid evidence is required.

Finally, genomic alterations may provide additional clues for treatment options. Although several databases (45) and scales (46) based on molecular targets have recently been released to guide target therapies for malignancies, evidence for rare tumors is lacking due to the rarity of morbidity. Still, case reports have shown that for rare tumors with actionable molecular alterations, targeted treatment would deliver clinical benefits (47, 48). Back to this case, both *PTEN* p.V275G and *TP53* p.T81Nfs*64 were classified as having uncertain clinical relevance according to previous literatures, and there are currently no approved drugs targeting *PTEN* or *TP53* mutations, with only preclinical attempts. *TSC2* mutation, which could contribute to the activation of the PI3K pathway, may be targeted by mTOR inhibitor everolimus in noncancerous diseases such as tuberous sclerosis (49, 50). On the other hand, somatic *PIK3CA* p.H1047R has become a targetable alteration for advanced breast cancer patients since the successful clinical trial of SOLAR-1 and the final approval of alpelisib (51). Thus, the *PIK3CA* mutation detected in our patient may indicate potential sensitivity to alpelisib. Nevertheless, it has also been reported that fulvestrant failed to deliver antiproliferative effects on a patient-derived PBOS cell line harboring a *PIK3CA* mutation (38). Furthermore, a patient-derive xenograft (PDX) model of PBOS has lately been reported, which offered an *in vivo* platform for the investigation of genome-informed treatment strategies (52). Hence, to further confirm the efficacy of anti-*PIK3CA* antigens for PBOS, preclinical models may provide more information.

## Conclusion

Primary breast osteosarcoma is a rare malignant tumor with divergence regarding its histogenesis, diagnosis, and management. In addition, as our case presented, a history of ipsilateral breast carcinoma could make the dilemma even worse. A thorough imaging review and meticulous pathological evaluation would be helpful to find the best plan of treatment. Moreover, complementary genomic approaches would also help us better understand its intrinsic features, even giving the opportunity for genome-informed targeted therapy for PBOS. Given the limited available data to guide management, further clinical and translational research is needed to optimize the treatment of this aggressive disease. Meanwhile, reporting each case and publishing them would be beneficial in gathering more information and offering collective efforts for finally managing this rare malignancy.

## Data availability statement

Data presented in this paper are available upon request.

## Ethics statement

Written informed consent was obtained from the individual(s) for the publication of any potentially identifiable images or data included in this article.

## Author contributions

SZ and JW were responsible for case management. SZ, JW, and HW contributed to the case review. XF conducted the pathological review. LL performed the targeted gene panel test. HW was responsible for the genomic evaluation. SZ and HW were the principal writers of the manuscript. JW and XF reviewed and provided valuable insight in the preparation of the paper. All authors contributed to the article and approved the submitted version.

## Supplementary material

Supplementary Figure 1 Timeline. Diagnosis and treatment timeline of this case.
